# Are hip fracture patients with high or low body mass index at higher risk of missed care? A cohort study

**DOI:** 10.1002/nop2.1687

**Published:** 2023-02-23

**Authors:** Nanna Sofie Astrup Pedersen, Inger Mechlenburg, Pia Kjær kristensen

**Affiliations:** ^1^ Department of Orthopaedics Aarhus University Hospital Aarhus Denmark; ^2^ Department of Clinical Medicine Aarhus University Aarhus Denmark

**Keywords:** body mass index, frail elderly, health care disparities, hip fractures, missed care, nurses, nursing, process measures, quality of health care

## Abstract

**Aim:**

To examine whether patients' body mass index is associated with missed hip fracture care consistent with national guideline‐recommended care.

**Design:**

A nationwide, population‐based cohort study using prospectively collected data from the Danish Multidisciplinary Hip Fracture Registry.

**Methods:**

The study population consisted of 39,835 patients ≥65 years admitted with a hip fracture and discharged between 1st of January 2012 and 29th of November 2017. National guideline‐recommended care consists of preoperative optimization, early surgery, mobilization within 24 h, basic mobility assessment, nutrition screening, post‐discharge rehabilitation program, and osteoporotic and fall prophylaxis. We used binomial regression to estimate the relative risk for the fulfilment of the individual measures with 95% confidence interval. Multiple imputation method was applied to handle missing values of body mass index.

**Results:**

The overall fulfilment of the individual measures ranged from 43% for pre‐operative optimization to 95% for receiving a post‐discharge rehabilitation program. The obese patients had a lower fulfilment of surgery within 36 h compared to patients with normal weight. No differences in fulfilment of the other measures were found. However, patients with missing data on body mass index had the highest risk of missed care. In conclusion, patients with missing BMI values had the highest risk of missed care. The obese patients had a slightly higher risk of long waiting times for surgery than normal‐weighted patients.

**No Patient or Public Contribution:**

This study was done based on population‐based data from medical registries and data was analysed by the authors only.

## 1 INTRODUCTION

Ensuring nursing care and patient safety is a major challenge facing healthcare systems today due to unprecedented challenges with increasing life expectancy and a concomitant increase in the prevalence of chronic diseases, multi‐morbidity, and frailty, resulting in greater demands of care. A mismatch between the level of nurse staffing, system demand, and shrinking budgets may compound challenges. Missed care, defined as any aspect of care that is omitted or delayed in part or in whole, is therefore receiving increased attention (Griffiths et al., 2018). However, previous studies on missed care have relied exclusively on nurse self‐reporting (Griffiths et al., 2018). Patients with hip fractures represent some of the frailest patients admitted to hospitals and may be at high risk of missed care (Frandsen et al., 2021). Several clinical quality databases for hip fracture care have been established in European countries with the aim of consistent evidence‐based hip fracture care. Although clinical quality databases have existed for many years, evidence has mainly focused on differences in patient outcomes like mortality at the hospital level. Few studies have examined potential patient‐related disparities in missed care. Denmark is an ideal setting for using routine data for longitudinal research to investigate missed care among patients with hip fractures, due to nationwide clinical quality databases and health care registries holding unique, detailed information on provided care, the prognostic profile of the patients, and patient outcomes. In this study, we, therefore, examined whether patients' BMI is associated with missed hip fracture care according to national guideline‐recommended care.

### 1.1 Background

Hip fracture is the leading cause of fall‐related mortality in elderly people with a mortality rate of 30% within the first year (Pedersen et al., 2017). Furthermore, patients with hip fractures have a high risk of postoperative complications (Goh et al., 2020) and reduced quality of life (Griffin, 2015). Among the survivors, only 50% returned to their previous functional capacity (Griffin, 2015). According to previous studies, unwarranted variation in patients' clinical outcomes exists (Kristensen et al., 2016). International and national clinical guidelines reflecting evidence‐based recommendations for fundamental care have therefore been developed to limit deviations and ensure a fast return to pre‐fracture functional capacity and thereby a better prognosis. Adherence to the guideline‐recommended care in routine clinical settings is in England, Ireland, and Denmark continuously monitored in clinical quality databases through the process performance measures: preoperative optimization, early surgery and mobilization, nutrition screening, pain assessment, rehabilitation, and prevention initiatives for recurrent fractures to ensure all patients fundamental care (NICE, 2011; Voeten et al., 2018). Fulfilment of these process performance measures is associated with lower mortality and readmission risk (Kristensen et al., 2016; Nielsen et al., 2009). However, not all patients with hip fractures receive the recommended fundamental care. Patients of older age or patients with comorbidities are associated with a lower chance of getting mobilized within 24 h after surgery (Kristensen et al., 2017; Schrøder et al., 2022). This may be related to the fact that it is more challenging to offer frail patients fundamental care needs. Providing fundamental care to patients with either high or low BMI may also require extra resources e.g. mobilization of an overweight or obese patient often requires more staff, assistive devices, time, and space (Patienter & Nelbom, 2007). Furthermore, being underweight is a predictor of obtaining suboptimal care in an acute setting (Hyldgård et al., 2021). We, therefore, hypothesized that patients with hip fractures who are underweight or obese have a higher risk of missed care compared to patients with hip fractures being normally weighted.

## 2 THE STUDY

### 2.1 Aim

This study aimed to investigate whether patients with a hip fracture who are underweight or obese, are at higher risk of missed care consistent with national guideline‐recommended care.

### 2.2 Design

A population‐based cohort study was conducted in Denmark, a country with 5.8 mills. inhabitants with tax‐supported healthcare. This study is reported according to the STROBE statement for observational studies.

### 2.3 Participants

**FIGURE 1:**
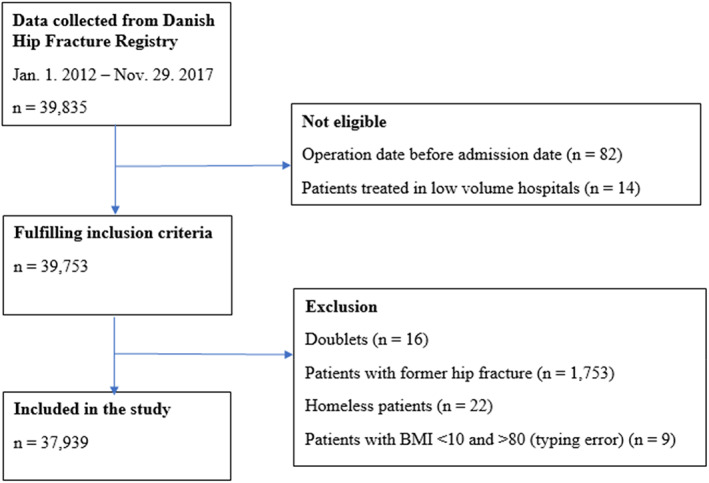
Flowchart illustrating the sampling, in‐ and exclusion process of the study population.

Data from the Danish Multidisciplinary Hip Fracture Registry (DMHFR) including data from the Danish National Patient Registry (DNPR) and the Civil Registration System (CRS) were used to identify patients ≥65 years old with a hip fracture and a discharge date between 1st of January 2012 and 29th of November 2017 (*n* = 39,835). Patients with a registered surgery date before the date of admission were excluded (*n* = 82) as well as patients coded as treated in hospital departments with fewer than 10 hip fracture patients per year (*n* = 14). Doublets (*n* = 16) and patients with more than one hip fracture during the study period were only included by their first admission (*n* = 1753). Furthermore, homeless patients (*n* = 22) and patients who were misclassified with a BMI <10 and > 80 (*n* = 9) were excluded. Our study cohort, therefore, included 37,939 patients (Figure 1).

### 2.4 Data collection

DMHFR is the clinical quality database for hip fracture care in Denmark and was established in 2003 to document and improve the quality of care in a hospital setting among hip fracture patients. The registry contains information about the fulfilment of recommended care and sociodemographic and biomedical characteristics, including BMI. Data are collected from the hospital departments treating hip fracture patients. DNPR was established in 1976 and contains data on hospital admissions, main and secondary diagnoses as well as diagnostic and surgical procedures. CRS was established in 1968 and contains records on vital status (date of death or emigration) for the entire Danish population with daily updates. Every Danish citizen is assigned a unique civil register number by CRS.

#### 2.4.1 Patient characteristics

Age was divided into three age groups 65–74, 75–84, ≥85 years old in line with studies investigating similar populations (Kristensen et al., 2017). Type of housing was classified as patients living in their own house, patients living in their own home affiliated to an institution, patients living at an institution, and unknown according to criteria used at the DMHFR. The comorbidity was measured using Charlson Comorbidity Index (CCI) (de Groot et al., 2003). The CCI score was divided into 0, 1, 2, or + 3 points (Schrøder et al., 2022). The fracture diagnosis is in DMHFR coded as medial, pertrochanteric, and subtrochanteric fracture. In this study, the two diagnoses of pertrochanteric and subtrochanteric fracture were collapsed as recommended by a study investigating the validity of hip fracture diagnoses in DMFHR and DNPR (Hjelholt et al., 2020).

#### 2.4.2 Body mass index

BMI value was calculated by weight in kilograms (kg) divided by the square of height in meters (m). Height and weight were measured by staff or self‐reported by patients. The BMI values were categorized according to the definition of WHO: Underweight (BMI <18.5 kg/m^2^), normal weight (BMI 18.5–24.9 kg/m^2^), overweight (BMI 25–29.9 kg/m^2^), and obese (BMI >30 kg/m^2^).

#### 2.4.3 Missed care

The risk of missed care was monitored through the fulfilment of ten process performance measures for pre‐, per‐, and postoperative care consistent with national guidelines recommended care (NICE, 2011; Voeten et al., 2018). The process performance measures included a surgical specialist assessment of the patient within 4 h after admission for planning pre‐operative optimization and early surgery within 24 or 36 h from the time of admission. Postoperatively, the patient has to be mobilized within 24 h from bed rest to walking or rest in a chair and having assessed their nutrition status and basic mobility using Cumulated Ambulation Score (CAS) (Foss et al., 2006). Finally, the patient must receive a post‐discharge rehabilitation plan, anti‐osteoporotic, and fall prophylaxis.

The recommendations and number of process performance measures have changed over time due to changes in evidence and clinical practice. See Table 1 for more details. Furthermore, some patients were excluded from the analysis. Patients who died under admission were deemed ineligible for measurement of basis mobility at discharge, for post‐discharge rehabilitation plan as well as initiation of treatment with anti‐osteoporotic medications and fall prophylaxis. Likewise, depending on identified contraindications (e.g., dementia or moribund patients), hospital staff could classify the patient as ineligible for the process performance measures mentioned above, as well as early mobilization. Therefore, the number of patients assessed in the analysis of the individual process performance measures varied.

**TABLE 1:** Year overview of availability of process performance measures and their fulfilment.

Process performance measures	2012	2013	2014	2015	2016	2017	Overall fulfilment
	% (*n*)	% (*n*)	% (*n*)	% (*n*)	% (*n*)	% (*n*)	% (fulfilment/relevant for the indicator)
Preoperative optimization							
Fulfilled	N/A	N/A	N/A	30% (1934)	47% (2993)	54% (2865)	43% (7792/18,135)
Not fulfilled				70% (4483)	53% (3383)	46% (2472)	
Not relevant				0	0	5	
Surgery delay < 24 t							
Fulfilled	N/A	N/A	N/A	67% (4327)	69% (4381)	64% (3392)	67% (12,100/18,135)
Not fulfilled				33% (2090)	31% (1995)	37% (1950)	
Not relevant				0	0	0	
Surgery delay < 36 t							
Fulfilled	N/A	N/A	N/A	84% (5402)	85% (5421)	83% (4431)	84% (15,254/18,135)
Not fulfilled				16% (1015)	15% (955)	17% (911)	
Not relevant				0	0	0	
Early mobilization							
Fulfilled	77% (5057)	77% (5201)	76% (4922)	49% (3138)	66% (4184)	68% (3628)	74% (26,130/37,939)
Not fulfilled	17% (1096)	15% (1009)	17% (1120)	45% (2853)	27% (1733)	27% (1418)	
Not relevant	7% (425)	7% (512)	7% (462)	7% (426)	7% (459)	6% (296)	
BMA prior to fracture							
Fulfilled	N/A	50% (3390)	79% (5143)	91% (5811)	92% (5851)	92% (4914)	87% (25,109/31,361)
Not fulfilled		13% (843)	21% (1361)	9% (606)	8% (525)	8% (428)	
Not relevant		37% (2489)	0	0	0	0	
BMA at discharge							
Fulfilled	75% (4911)	80% (5356)	81% (5285)	87% (5563)	88% (5589)	87% (4619)	88% (31,323/37,939)
Not fulfilled	19% (1228)	14% (918)	13% (844)	8% (512)	7% (446)	9% (478)	
Not relevant	7% (439)	7% (448)	6% (375)	5% (342)	5% (341)	5% (245)	
Initiation of nutrition plan							
Fulfilled	N/A	N/A	N/A	78% (5009)	85% (5392)	84% (4467)	82% (14,868/18,135)
Not fulfilled				22% (1408)	15% (984)	16% (875)	
Not relevant				0	0	0	
Medical prophylaxis							
Fulfilled	87% (5720)	89% (5947)	85% (5519)	84% (5360)	86% (5471)	85% (4559)	91% (32,576/37,939)
Not fulfilled	6% (419)	5% (327)	9% (610)	11% (715)	9% (565)	10% (538)	
Not relevant	7% (439)	7% (448)	6% (375)	5% (342)	5% (340)	5% (245)	
Fall prophylaxis							
Fulfilled	82% (5414)	83% (5594)	82% (5303)	81% (5174)	84% (5350)	84% (4511)	88% (31,346/37,939)
Not fulfilled	11% (725)	10% (680)	13% (826)	14% (901)	11% (686)	11% (586)	
Not relevant	7% (439)	7% (448)	6% (375)	5% (342)	5% (340)	5% (245)	
Initiation of rehabilitation plan							
Fulfilled	86% (5660)	87% (5824)	86% (5610)	86% (5531)	88% (5616)	90% (4828)	95% (33,069/37,939)
Not fulfilled	3% (210)	3% (213)	4% (265)	7% (437)	5% (294)	5% (263)	
Not relevant	11% (708)	10% (685)	10% (629)	7% (449)	7% (466)	5% (251)	

Abbreviations: BMA, Basic mobility assessment; N/A, Not available.

### 2.5 Data analysis

We described the overall study population and patients in the different BMI groups using summary statistics. The proportions of patients who receive the fundamental care recommendations were calculated overall and for each BMI group. Binomial regression was used to estimate the relative risk (RR) with 95% confidence interval (95% CI) of the association between the BMI group and fulfilment of the individual process performance measures. In the regression analysis, the multiple imputation method was applied to handle missing data on BMI and type of housing, using information available on patient characteristics presented in Table 2, hospital departments, and outcomes applicable in the entire study period. We generated 20 imputed datasets by chained equations based on the missing at random assumption (Sterne et al., 2009). The binomial regression analyses were also performed as complete case analyses. Data management and analysis were performed using STATA version 15.1 (StataCorp LP, College Station).

**TABLE 2:** Characteristics of patients with hip fracture according to BMI group or missing data on BMI.

Patient characteristics	Overall 100% (*N* = 37,939)	Underweight 8% (*n* = 3172)	Normal weight 46% (*n* = 17,568)	Overweight 22% (*n* = 8246)	Obese 7% (*n* = 2520)	Missing data on BMI 17% (*n* = 6433)
Sex						
Women	70% (26.590)	86% (2732)	72% (12,585)	62% (5096)	68% (1708)	70% (4469)
Men	30% (11.349)	14% (440)	28% (4983)	38% (3150)	32% (812)	30% (1964)
Age group						
65–74 years	21% (7786)	19% (588)	19% (3333)	22% (1820)	29% (730)	20% (1315)
75–84 years	36% (13,750)	34% (1090)	35% (6142)	40% (3257)	43% (1091)	34% (2170)
≥85 years	43% (16,403)	47% (1494)	46% (8093)	38% (3169)	28% (699)	46% (2948)
Housing						
Own home	69% (26,272)	72% (2277)	74% (12,984)	76% (6291)	80% (2010)	42% (2710)
Own home affiliated to an institution	5% (2053)	6% (182)	6% (1050)	6% (475)	5% (130)	3% (216)
Institution	17% (6501)	20% (617)	18% (3101)	16% (1285)	13% (323)	18 (1175)
Unknown	8% (3113)	3% (96)	3% (433)	2% (195)	2% (57)	36% (2332)
CCI score						
0: No comorbidity	37% (13,953)	35% (1095)	39% (6768)	38% (3104)	34% (861)	33% (2125)
1: Low comorbidity	23% (8834)	25% (786)	23% (4118)	22% (1817)	22% (556)	24% (1557)
2: Moderate comorbidity	18% (6711)	19% (600)	18% (3071)	18% (1439)	16% (409)	19% (1192)
+3: High comorbidity	22% (8441)	22% (691)	21% (3611)	23% (1886)	28% (694)	24% (1559)
Type of fracture						
Medial	55% (20,797)	50% (1582)	55% (9640)	57% (4685)	54% (1361)	55% (3529)
Lateral	45% (17.142)	50% (1590)	45% (7928)	43% (3.561)	46% (1159)	45% (2904)

*Note*: Underweight: BMI < 18.5 kg/m^2^; normal weight: BMI 18.5–24.9 kg/m^2^; overweight: BMI 25–29.9 kg/m^2^; obese: BMI ≥30 kg/m^2^.

Abbreviations: BMI, Body mass index; CCI, Charlson Comorbidity Index.

### 2.6 Validity, reliability, and rigour

Misclassification, when data are collected during routine clinical work in a large number of settings, is a possibility. However, data validity is highly prioritized in the DMHFR, which has detailed data definitions and auditing of data quality performed continuously at local and regional levels and annually at the national level. Furthermore, the misclassification of the fulfilment of process performance measures would most likely be unrelated to the BMI group since registration of BMI was done without knowing the aim of this study.

## 3 RESULTS

### 3.1 Patient characteristics

We identified 37,939 patients with hip fractures with a discharge date between 1st of January 2012 and 29th of November 2017. The mean age of the study population was 83 (range 65–107) years and was predominantly female (70%) and mainly living in their own home (69%). Most of the patients were normally weighted (46%) and 17% had missing data on BMI. See Table 2 for characteristics of patients with hip fractures according to BMI group or missing BMI. There was a slight trend of increase in the proportion of men as BMI value increases. Compared to patients with normal weight, the obese patients were on average younger, living in their own houses, and had more comorbidities. Patients with underweight had similar characteristics as the patients with normal weight. Among the patients with missing data on BMI, nearly half were ≥ 85 years old and 37% had missing data on the type of housing.

### 3.2 Risk of missed care

Table 3 provides the descriptive statistics of the fulfilment of process performance measures according to BMI group including patients with missing data on BMI. The overall fulfilment of the process performance measures in the hip fracture population ranged from 43% for preoperative optimization to 95% for receiving a post‐discharge rehabilitation plan. Patients with missing data on BMI had the lowest fulfilment of the process performance measures ranging from 22% to 90%. Table 4 presents the fulfilment of process performance measures according to BMI group using multiple imputation method. The analysis showed that patients who were either underweight or overweight had the same risk of missed care compared to normal‐weight patients. The RR for fulfilment for underweight patients varied from 0.90 to 1.10 with an absolute difference in fulfilment of 2% between underweighted and normal‐weighted patients. For overweight patients, the RR varied from 0.96 to 1.09 with an absolute difference in fulfilment between the groups of 1%. The obese patients had a lower fulfilment of surgery within 36 h compared to normal‐weight patients (82% vs. 85%) corresponding to a RR of 0.85 (95% CI 0.72–0.998). The complete case analysis provided results with only minor BMI‐related differences in risk of missed care and with similar trends as the imputation analyses. See Table S1 in the Supporting Information for complete case analysis of fulfilment of process performance measures according to BMI group. Some of the differences between underweight patients and patients with normal weight reached statistical significance, although the absolute differences were minor. No differences were found in the proportion of patients considered eligible for the individual processes in patients with data on BMI. See Table S2 in the Supporting Information for more details.

**TABLE 3:** Fulfilment of process performance measures according to BMI group including patients with missing data on BMI.

Process performance measures	Overall	Normal weight (ref.)		Underweight		Overweight		Obese		Missing data on BMI	
	% (*n*)	% (*n*)	RR	% (*n*)	RR (95% CI)	% (*n*)	RR (95% CI)	% (*n*)	RR (95% CI)	% (*n*)	RR (95% CI)
Preoperative optimization	43% (7792)	45% (3842)	1.00	46% (650)	0.96 (0.91–1.03)	48% (1930)	1.00 (0.96–1.04)	50% (639)	1.03 (0.97–1.10)	22% (731)	0.45 (0.42–0.48)
Surgery delay < 24 t	67% (12,100)	68% (5438)	1.00	70% (991)	1.04 (1.0008–1.08)	67% (2714)	0.99 (0.97–1.02)	67% (866)	0.99 (0.95–1.03)	62% (2091)	0.91 (0.88–0.94)
Surgery delay < 36 t	84% (15,254)	85% (6851)	1.00	87% (1217)	1.01 (0.99–1.04)	85% (3411)	0.99 (0.98–1.01)	83% (1069)	0.97 (0.94–0.99)	80% (2706)	0.93 (0.92–0.95)
Early mobilization	74% (26,130)	78% (12,888)	1.00	77% (2242)	0.98 (0.96–1.00)	78% (6094)	1.00 (0.99–1.02)	76% (1791)	0.98 (0.95–0.999)	54% (3115)	0.69 (0.67–0.71)
BMA prior to fracture	87% (25,109)	91% (12,012)	1.00	91% (2153)	1.00 (0.98–1.01)	91% (5818)	1.00 (0.99–1.01)	92% (1807)	1.01 (0.99–1.02)	67% (3319)	0.74 (0.72–0.75)
BMA at discharge	88% (31,323)	91% (15,060)	1.00	90% (2638)	1.00 (0.98–1.01)	90% (7157)	1.00 (0.99–1.00)	90% (2171)	0.99 (0.98–1.01)	74% (4297)	0.82 (0.80–0.83)
Initiation of nutrition plan	82% (14,868)	89% (7172)	1.00	88% (1231)	0.98 (0.96–0.999)	90% (3637)	1.01 (1.00–1.02)	91% (1171)	1.01 (0.99–1.03)	49% (1657)	0.55 (0.53–0.57)
Medical prophylaxis	91% (32,576)	96% (16,037)	1.00	97% (2847)	1.01 (1.002–1.02)	96% (7630)	1.00 (0.99–1.00)	97% (2335)	1.00 (0.99–1.01)	64% (3727)	0.66 (0.65–0.68)
Fall prophylaxis	88% (31,346)	93% (15,537)	1.00	94% (2738)	1.00 (0.99–1.01)	94% (7446)	1.00 (1.00–1.01)	94% (2268)	1.00 (0.99–1.02)	58% (3357)	0.62 (0.60–0.63)
Rehabilitation plan	95% (33,069)	96% (15,563)	1.00	96% (2715)	0.99 (0.99–1.00)	96% (7446)	1.00 (0.99–1.01)	97% (2275)	1.00 (0.99–1.01)	90% (5070)	0.93 (0.92–0.94)

*Note*: Underweight: BMI < 18.5 kg/m^2^; normal weight: BMI 18.5–24.9 kg/m^2^; overweight: BMI 25–29.9 kg/m^2^; obese: BMI ≥30 kg/m^2^.

Abbreviations: BMI, Body mass index; BMA, Basic mobility assessment; CI, Confidence interval; RR, Relative risk.

**TABLE 4:** Fulfilment of process performance measures according to BMI group. Proportions and RR estimated using multiple imputation method.

Process performance measures	Normal weight (ref.)		Underweight		Overweight		Obese	
	%	RR (95% CI)	%	RR (95% CI)	%	RR (95% CI)	%	RR (95% CI)
Preoperative optimization	43%	1.00	41%	0.94 (0.84–1.05)	43%	1.01 (0.95–1.09)	45%	1.08 (0.96–1.21)
Surgery delay < 24 t	67%	1.00	69%	1.10 (0.98–1.23)	66%	0.98 (0.91–1.06)	66%	0.97 (0.85–1.10)
Surgery delay < 36 t	84%	1.00	85%	1.06 (0.90–1.24)	84%	0.96 (0.87–1.07)	82%	0.85 (0.72–0.998)
Early mobilization	74%	1.00	73%	0.94 (0.86–1.03)	74%	1.01 (0.95–1.08)	73%	0.92 (0.84–1.02)
BMA prior to fracture	87%	1.00	87%	0.97 (0.85–1.12)	87%	1.03 (0.93–1.13)	88%	1.07 (0.91–1.25)
BMA at discharge	88%	1.00	87%	0.96 (0.84–1.10)	88%	0.98 (0.90–1.07)	87%	0.94 (0.82–1.08)
Initiation of nutrition plan	82%	1.00	80%	0.90 (0.76–1.05)	83%	1.09 (0.98–1.21)	83%	1.12 (0.93–1.34)
Medical prophylaxis	91%	1.00	92%	1.08 (0.92–1.27)	91%	0.98 (0.89–1.09)	91%	1.02 (0.84–1.23)
Fall prophylaxis	88%	1.00	88%	1.00 (0.88–1.14)	88%	1.04 (0.95–1.13)	88%	1.03 (0.88–1.21)
Initiation of rehabilitation plan	95%	1.00	95%	0.92 (0.76–1.10)	95%	1.02 (0.90–1.16)	96%	1.08 (0.87–1.33)

*Note*: Underweight: BMI < 18.5 kg/m^2^; normal weight: BMI 18.5–24.9 kg/m^2^; overweight: BMI 25–29.9 kg/m^2^; obese: BMI ≥30 kg/m^2^.

Abbreviations: BMI, Body mass index; BMA, Basic mobility assessment; CI, Confidence interval; RR, Relative risk.

## 4 DISCUSSION

In this nationwide study of missed care among 37,939 elderly patients with hip fractures, we found a major risk of missed hip fracture care. Patients with missing BMI values had the highest risk of missed care. Patients with underweight or overweight were not at increased risk of missed care compared to patients, who were normal weight. The obese patients had a slightly higher risk of long waiting times for surgery than normal‐weight patients.

To our knowledge, no prior studies have examined whether patients suffering from hip fractures with underweight or overweight were at increased risk of missed hip fracture care. In contrast to our hypothesis, we found a similar occurrence of missed care among patients with either normal weight, underweight or overweight. However, we found that patients with missing BMI values had a higher risk of missed care. Poor documentation of nursing care has previously been reported as a common missed care activity with implications for patient outcomes (Kristensen et al., 2016). Fulfilment of the fundamental hip fracture care process performance measures in this study has previously been shown to be associated with lower mortality and readmission risk (Nielsen et al., 2009). Our findings, therefore, support the previous findings of a higher risk of missed care among patients for whom poor documentation has been practiced.

The obese patients had a slightly higher risk of surgery delay compared to patients with normal weight. It could be a sign of insufficient capacity due to organizational factors, as it may be more complicated to anaesthetise and operate on obese patients (Akinleye et al., 2018). However, it could also be related to more comorbidities among obese patients compared to patients with normal weight (Bergeron et al., 2006). Early surgery is associated with reductions in perioperative mortality and postoperative complications and is therefore of great importance (Chow et al., 2012). Unfortunately, there is a lack of research investigating how to identify patients for whom the need for preoperative optimization can justify a delay in time for surgery. The need for efficient and focused preoperative risk assessment and optimization is of great importance to achieve improved outcomes (Nicholas, 2014). Nevertheless, only 50% of obese patients in the study population had received preoperative optimization. The waiting time was therefore not always explained by preoperative optimization.

Interestingly, the obese patients in our study had the same chance of mobilization within 24 h postoperative as patients with normal weight. This contrasts with a previous study from the United States which has found delayed mobilization of obese patients (Bryant et al., 2019).

Overall, the prevalence of missed care was common among patients with hip fractures and there is therefore a need for intervention with a broader perspective than BMI. A focus on early and structured planning of post‐operative care could support the staff in the implementation of hip fracture care according to recommendations, e.g. analysis of possible barriers for early mobilization or planning of nutritional support for the underweight patient, so interventions could begin immediately after surgery.

### 4.1 Limitations

This study is population‐based and contributes to a large sample size, with prospectively collected data and complete follow‐up which helped minimize the risk of selection and information bias. Data on BMI by patient report or measured by staff were considered valid information which minimize the misclassification of exposure (Bell et al., 2013). As missing data on BMI also meant a remarkably high risk of missed care compared to the BMI groups, the results could be biased if only patients with complete data were included. To handle this, the multiple imputation method was therefore applied in the binary regression analysis using patient characteristics, hospital departments, and outcomes to impute data on BMI. The outcome in the imputation model was used since the outcome carries information about the missing values of the predictor, and this information must therefore be used (Sterne et al., 2009). Missing data on the type of housing were imputed as well in a chain since missing data on the type of housing were correlated with missing data on BMI. See Table S3 in the Supporting Information for more details. The results had the narrowest confidence intervals when the imputation was conducted using chained equations with BMI and type of housing compared to univariate analysis, imputing only BMI.

As only eligible patients were included, no adjustment for covariates was conducted. For the process, performance measures *early mobilization*, *basic mobility assessment*, *medical and fall prophylaxis*, and *initiation of a rehabilitation plan*, the hospital staff could consider the patients ineligible because of e.g., weakness, dementia, or moribund patients. The eligibility for the process performance measures was determined by the hospital staff and assessment may differ based on a different level of experience and working environment in the different hospital departments. However, we did not find differences in the proportion considered eligible for the individual processes across BMI groups. Patients who died during admission were registered as ineligible and therefore excluded from the analysis.

## 5 CONCLUSION

In conclusion, we found a major risk of missed care among elderly patients with hip fractures in Denmark. Particularly patients with missing data on BMI had the highest risk of missed care and obese patients had a slightly higher risk of waiting for surgery than patients with normal weight. No differences in risk of missed care were found between patients with underweight or overweight compared to patients with normal weight. Improving the care for obese patients should therefore not be the sole target in future work. But a firm focus on nurses' prioritizing fundamental care will benefit all patients with hip fractures and prevent variations in clinical outcomes caused by missed care.

## AUTHOR CONTRIBUTIONS

The authors have no conflict of interests and meet the authorship requirements as stated; NSAP, IM and PKK conceived and designed the study. NSAP performed the analysis. NSAP, IM and PKK interpreted the data. NSAP wrote the initial draft. All authors critically revised the paper for important intellectual content and gave final approval of the published version.

## FUNDING INFORMATION

This research received no specific grant from any funding agency in the public, commercial, or not‐for‐profit sectors.

## CONFLICT OF INTEREST STATEMENT

The author reports no conflicts of interest.

## ETHICS STATEMENT

The study was approved by the Data Protection Agency, Central Region Denmark (Journal no. REDACTED). Informed consent from the participants is not needed for register‐based studies in the Northern Countries (Ludvigsson et al., 2015).

## Supporting information


Table S1:

Table S2:

Table S3:

## Data Availability

The data that support the findings of this study are available on request from the DMHFR after approval of the Data Protection Agency, Central Region Denmark. The data are not publicly available due to privacy and ethical restrictions.
