# Supplementary material for: Are hip fracture patients with high or low body mass index at higher risk of missed care? A cohort study
Source: Nurs Open. 2023 Feb 23;10(7):4452–60. doi: 10.1002/nop2.1687 (PMC10277429; doi:10.1002/nop2.1687)
Supplement: Supplementary file 1 — Table S1: [file NOP2-10-4452-s003.docx]

| **Table S1** Fulfillment of process performance measures according to BMI group as complete case | | | | | | | | | | | | | |
| --- | --- | --- | --- | --- | --- | --- | --- | --- | --- | --- | --- | --- | --- |
| **Process performance measures** | **Patients with BMI data, *n*** | **Normal weight (ref.)** | |  | | **Underweight** | |  | **Overweight** | |  | **Obese** | |
|  |  | **% (n)** | **RR (95% CI)** | |  | **% (n)** | **RR (95% CI)** |  | **% (n)** | **RR (95% CI)** |  | **%** | **RR (95% CI)** |
| **Preoperative optimization** | 18,135 | 48% (3,842) | 1.00 |  | | 46% (650) | 0.96 (0.91-1.03) |  | 48% (1,930) | 1.00 (0.96-1.04) |  | 50% (639) | 1.03 (0.97-1.10) |
| **Surgery delay <24t** | 18,135 | 68% (5,438) | 1.00 |  | | 70% (991) | 1.04 (1.0008-1.08) |  | 67% (2,714) | 0.99 (0.97-1.02) |  | 67% (866) | 0.99 (0.95-1.03) |
| **Surgery delay <36t** | 18,135 | 85% (6,851) | 1.00 |  | | 87% (1,217) | 1.01 (0.99-1.01) |  | 85% (3,411) | 0.99 (0.98-1.01) |  | 83% (1,069) | 0.97 (0.94-0.99) |
| **Early mobilization** | 37,939 | 78% (12,888) | 1.00 |  | | 77% (2,242) | 0.98 (0.96-1.00) |  | 78% (6,094) | 1.00 (0.99-1.02) |  | 76% (1,791) | 0.98 (0.95-0.999) |
| **BMA prior to fracture** | 31,361 | 91% (12,012) | 1.00 |  | | 91% (2,153) | 1.00 (0.98-1.01) |  | 91% (5,818) | 1.00 (0.99-1.01) |  | 92% (1,807) | 1.01 (0.99-1.02) |
| **BMA at discharge** | 37,939 | 91% (15,060) | 1.00 |  | | 90% (2,638) | 1.00 (0.98-1.01) |  | 90% (7,157) | 1.00 (0.99-1.00) |  | 90% (2,171) | 0.99 (0.98-1.01) |
| **Initiation of nutrition plan** | 18,135 | 89% (7,172) | 1.00 |  | | 88% (1,231) | 0.98 (0.96-0.999) |  | 90% (3,637) | 1.01 (1.00-1.02) |  | 91% (1,171) | 1.01 (0.99-1.03) |
| **Medical prophylaxis** | 37,939 | 96% (16,037) | 1.00 |  | | 97% (2,847) | 1.01 (1.002-1.02) |  | 96% (7,630) | 1.00 (0.99-1.00) |  | 97% (2,335) | 1.00 (0.99-1.01) |
| **Fall prophylaxis** | 37,939 | 93% (15,537) | 1.00 |  | | 94% (2,738) | 1.00 (0.99-1.01) |  | 94% (7,446) | 1.00 (1.00-1.01) |  | 94% (2,268) | 1.00 (0.99-1.02) |
| **Initiation of rehabilitation plan** | 37,939 | 96% (15,563) | 1.00 |  | | 96% (2,715) | 0.99 (0.99-1.00) |  | 96% (7,446) | 1.00 (0.99-1.01) |  | 97% (2,275) | 1.00 (0.99-1.01) |
| Underweight: BMI < 18.5 kg/m^2^; normal weight: BMI 18.5-24.9 kg/m^2^; overweight: BMI 25-29.9 kg/m^2^; obese: BMI ≥ 30 kg/m^2^  *BMI* Body mass index  *BMA* Basic mobility assessment  *RR* Relative risk  *CI* Confidence interval | | | | | | | | | |  |  |  |  |
