# Supplementary material for: Are hip fracture patients with high or low body mass index at higher risk of missed care? A cohort study
Source: Nurs Open. 2023 Feb 23;10(7):4452–60. doi: 10.1002/nop2.1687 (PMC10277429; doi:10.1002/nop2.1687)
Supplement: Supplementary file 2 — Table S2: [file NOP2-10-4452-s002.docx]

| **Table S2** Proportion of patients considered eligible for the individual processes according to BMI group  **,** | | | | | | | | | | | |
| --- | --- | --- | --- | --- | --- | --- | --- | --- | --- | --- | --- |
| **Process performance measures** | **Normal weight** | |  | **Underweight** | |  | **Overweight** | |  | **Obese** | |
|  | **% (n)** |  |  | **%** |  |  | **%** |  |  | **%** |  |
| **Early mobilization** | 5% (859) |  |  | 6% (183) |  |  | 5% (394) |  |  | 6% (150) |  |
| **BMA at discharge** | 0.2% (35) |  |  | 0.3% (8) |  |  | 0.2% (12) |  |  | 0.1% (2) |  |
| **Medical prophylaxis** | 0.2% (35) |  |  | 0.3% (8) |  |  | 0.2% (12) |  |  | 0.1% (2) |  |
| **Fall prophylaxis** | 0.2% (35) |  |  | 0.3% (8) |  |  | 0.2% (12) |  |  | 0.1% (2) |  |
| **Initiation of rehabilitation plan** | 3% (500) |  |  | 3% (99) |  |  | 3% (218) |  |  | 3% (63) |  |
| Underweight: BMI < 18.5 kg/m^2^; normal weight: BMI 18.5-24.9 kg/m^2^; overweight: BMI 25-29.9 kg/m^2^; obese: BMI ≥ 30 kg/m^2^  *BMI* Body mass index  *BMA* Basic mobility assessment | | | | | | | | | | | |
