# Supplementary material for: Are hip fracture patients with high or low body mass index at higher risk of missed care? A cohort study
Source: Nurs Open. 2023 Feb 23;10(7):4452–60. doi: 10.1002/nop2.1687 (PMC10277429; doi:10.1002/nop2.1687)
Supplement: Supplementary file 3 — Table S3: [file NOP2-10-4452-s001.docx]

| **Table S3** Prediction analysis of missing data on BMI | | | |
| --- | --- | --- | --- |
| Patient characteristics | **Missing BMI**  % (n) | **Available data on BMI**  % (n) | **OR for missing data on BMI**  Unadjusted OR (CI 95%) |
| **Sex** |  |  |  |
| Women | 69% (4,469) | 70% (22,122) | 1 |
| Men | 31% (1,964) | 30% (9,385) | 1.03 (0.98-1.08) |
| **Age group** |  |  |  |
| 65-74 years | 20% (1,315) | 21% (6,471) | 1 |
| 75-84 years | 34% (2,170) | 37% (11,580) | 0.93 (0.88-0.99) |
| ≥85 years | 46% (2,948) | 43% (13.455) | 1.06 (1.003-1.13) |
| **Type of housing** |  |  |  |
| Own home | 42% (2,710) | 75% (23,562) | 1 |
| Own home affiliated to an institution | 3% (216) | 6% (1,837) | 1.02 (0.89-1.16) |
| Institution | 18% (1,175) | 17% (5,326) | 1.75 (1.65-1.87) |
| Unknown | 36% (2.332) | 3% (781) | 7.26 (6.97-7.57) |
| **CCI score** |  |  |  |
| 0: No comorbidity | 33% (2,125) | 38% (11,828) | 1 |
| 1: Low comorbidity | 24% (1,557) | 23% (7,277) | 1.16 (1.09-1.23) |
| 2: Moderate comorbidity | 19% (1,192) | 18% (5.519) | 1.17 (1.09-1.24) |
| +3: High comorbidity | 24% (1.559) | 22% (6.882) | 1.21 (1.14-1.29) |
| **Type of fracture** |  |  |  |
| Medial | 55% (3,529) | 55% (17,268) | 1 |
| Lateral | 45% (2,904) | 45% (14,238) | 1 (0.95-1.04) |
| **Hospital department** |  |  |  |
| 1 | 17% (91) | 83% (449) |  |
| 2 | 23% (492) | 77% (1,658) |  |
| 3 | 16% (6) | 84% (31) |  |
| 4 | 6% (107) | 94% (1,660) |  |
| 5 | 9% (156) | 91% (1,658) |  |
| 6 | 6% (101) | 94% (1,484) |  |
| 7 | 15% (231) | 85% (1,292) |  |
| 8 | 2% (34) | 98% (2,048) |  |
| 9 | 6% (73) | 94% (1,139) |  |
| 10 | 13% (457) | 87% (3,099) |  |
| 11 | 16% (222) | 84% (1,161) |  |
| 12 | 14% (293) | 87% (1,873) |  |
| 13 | 11% (197) | 89% (1,556) |  |
| 14 | 9% (32) | 91% (326) |  |
| 15 | 51% (1,062) | 49% (1,032) |  |
| 16 | 29% (842) | 71% (2,071) |  |
| 17 | 40% (903) | 60% (1,361) |  |
| 18 | 3% (78) | 97% (2,495) |  |
| 19 | 6% (22) | 94% (340) |  |
| 20 | 28% (664) | 73% (1,750) |  |
| 21 | 11% (184) | 89% (1,504) |  |
| 22 | 11% (186) | 89% (1,519) |  |
| Total | 17% (6,433) | 83% (31,506) |  |
| *BMI* Body mass index  *CCI* Charlson morbidity index  *OR* Odds ratio |  |  |  |
